# Emotional modulation of statistical learning in visual search

**DOI:** 10.3389/fcogn.2024.1404112

**Published:** 2024-06-13

**Authors:** Artyom Zinchenko, Afton M. Bierlich, Markus Conci, Hermann J. Müller, Thomas Geyer

**Affiliations:** ^1^Department Psychologie, Ludwig-Maximilians-Universität München, München, Germany; ^2^NeuroImaging Core Unit Munich (NICUM), Ludwig-Maximilians-Universität München, München, Germany; ^3^Klinik und Poliklinik für Psychiatrie und Psychotherapie, Ludwig Maximilian University of Munich (LMU) Klinikum, LMU München, München, Germany; ^4^Munich Center for Neurosciences—Brain and Mind, Ludwig-Maximilians-Universität München, München, Germany

**Keywords:** visual search, statistical learning, contextual cueing, emotion and motivation, passive and active search strategies

## Abstract

**Introduction:**

Visual search is facilitated when participants encounter targets in repeated display arrangements. This “contextual-cueing” effect is attributed to incidental learning of spatial distractor-target relations, which subsequently guides visual search more effectively toward the target location. Conversely, behaviorally significant, though task-irrelevant, negative emotional stimuli may involuntarily capture attention and thus hamper performance in visual search. This raises the question of how these two attention-guiding factors connect.

**Methods:**

To this end, we investigated how an emotionally alerting stimulus induced by different classes of emotional (face, scene) pictures prior to the search task relates to memory-related plasticity. We tested 46 participants who were presented with repeated and non-repeated search layouts, preceded at variable (50, 500, 1,000 ms) intervals by emotional vs. neutral faces or scenes.

**Results:**

We found that contextual learning was increased with emotional compared to neutral scenes, which resulted in no contextual cueing was observed at all, while no modulation of the cueing effect was observed for emotional (vs. neutral) faces. This modulation occurred independent of the intervals between the emotional stimulus and the search display.

**Discussion:**

We conclude that emotional scenes are particularly effective in withdrawing attentional resources, biasing individual participants to perform a visual search task in a passive, i.e., receptive, manner, which, in turn, improves automatic contextual learning.

## Introduction

Humans display an impressive capability for extracting statistical regularities from previously encountered scenes. For example, finding a searched-for “target”, such as some item in the supermarket, is facilitated by finding it repeatedly at the same location within a predictable arrangement relative to other, surrounding items. In a comparable laboratory setting, observers are likewise faster in detecting a T-shaped target letter embedded in a set of L-shaped non-target, or distractor, letters when the spatial arrangement of the search items is repeated across trials. Such repeatedly encountered target-distractor arrangements, or “contexts”, come to guide visual search, “cueing” attention to the target location, even in the absence of participants' explicit knowledge about the repeated displays (Chun and Jiang, 1998). While such a contextual-cueing effect typically reveals a benefit to guide visual selective attention more effectively in search tasks (e.g., Wolfe, 2021), there is an ongoing controversy whether incidental learning of such target-distractor associations is also influenced by stimuli that signal negative emotional events and thus stimulate participants' psychological and physiological processing (for review, see, e.g., Dolcos et al., 2020).

Of particular relevance for the present work is a study by Zinchenko et al. (2020; see also Meyer et al., 2019), which found enhanced contextual facilitation of attentional guidance when the search items were presented superimposed on task-irrelevant background scenes that induced negative (as opposed to neutral) emotions, taken from the International Affective Picture System (IAPS; Lang et al., 2005). While this suggests that (negative) emotional experiences up-modulate contextual learning, the above-mentioned studies have presented emotional pictures only during the ongoing search task. This raises the question whether other emotion induction methods effectively modulate contextual learning as well. This question is important as prior work that also employed the contextual cueing paradigm found that negative emotional stimuli presented before the search task diminished (rather than enhanced) the reaction time (RT) contextual-facilitation effect (Kunar et al., 2014). However, this study actually used a specific mood induction scheme in which a set of 20 negative emotional pictures were all presented before the search task. The emotion-inducing stimuli may thus have elicited a rather persistent negative affective state, or “mood”, which may differ qualitatively from the transient behavioral and physiological “alarm” responses arising through the actual sensing of emotional information (Qiao-Tasserit et al., 2017). In this view, emotional stimuli may increase the cueing effect (Meyer et al., 2019; Zinchenko et al., 2020) when presented before each search trial, thus engaging more transient emotional responses, but may conversely also reduce cueing when the emotional stimuli induce a persistent change of the mood (Kunar et al., 2014). Furthermore, Kunar et al. (2014) used different types of emotional stimuli: faces vs. IAPS scenes, although they did not analyze their dependent measures separately for these stimulus classes. Both stimuli can reliably induce emotions (e.g., Thom et al., 2014), but the time course of recognizing or extracting emotional expressions differs between faces and scenes.

For instance, several EEG studies have demonstrated that recognition of emotional compared to neutral faces as indexed by effects at visual posterior electrodes is faster compared to (emotional vs. neutral) scenes, with a latency of about 120–170 ms for faces (e.g., Eimer and Holmes, 2007) and a latency of 220–280 ms for scenes (e.g., Thom et al., 2014). Moreover, processing differences between emotional and neutral stimuli were also found in the early visual cortex at relatively early time points and commencing at about 200 ms for faces, but only at later time points (>550 ms) for scenes when these stimuli served as task-irrelevant background distractors in an attention-demanding visual dot-detection task that used steady-state visual evoked potentials to measure attention deployment (Bekhtereva et al., 2015).

Based on these findings, in the present work, we systematically tested the effects of different classes of emotional—face and scene—stimuli that were presented at variable (50–1,000 ms) intervals before the search task that contained repeated and non-repeated layouts to account for likely processing differences between these stimuli and thus being able to maximize the effects that emotional faces and scenes may have on incidental contextual learning. On the basis of prior (own) findings (Zinchenko et al., 2020), we predicted increased contextual cueing with emotional over neutral stimuli, with these effects of emotions possibly becoming stronger with longer inter-stimulus intervals, that is, when the emotional recognition of both faces and IAPS scenes is fully effective (e.g., Bekhtereva et al., 2015).

An emotional modulation of contextual cueing may occur because the processing of negative emotional events, although task-irrelevant, soaks up attentional resources from the main task (Pessoa et al., 2002). However, while this may impair task performance (e.g., Bekhtereva et al., 2015, who found that signal-detection accuracy in their visual dot-detection task was lower with emotional relative to neutral faces/scenes), it may benefit incidental, i.e., automatic, contextual learning. This prediction is based on findings from earlier cueing studies, which demonstrated that contextual learning is reduced when participants engage in visual search in a stronger attentive, or active, fashion, compared to conditions where visual search is conducted more passively or intuitively (Lleras and Von Mühlenen, 2004). In this view, emotional materials may enhance contextual learning through an interference effect: negative emotional stimuli, signaling high behavioral relevance and are therefore prioritized in the competition for attentional processing. Consequently, fewer processing resources are left for the primary task. This may induce a bias to perform search in a more passive/receptive manner, thereby paradoxically enhancing the cueing effect (Lleras and Von Mühlenen, 2004).

To forecast the results, we found improved visual search of repeated compared to non-repeated letter search arrays. This effect was greater for (negative) emotional relative to neutral scenes, while contextual cueing was unaffected by emotional face stimuli. These difference between emotional and neutral scenes were evident at all intervals between the emotion-inducing pictures and the search displays. Given this, we suspect that emotional scenes and faces interfere with attentional resource allocation, as demonstrated in previous studies, though the specific stimuli may differ in their capacity to influence attentional processing and thus, in their ability to influence contextual learning.

## Methods

### Participants and setup

Forty six participants took part in the experiment (28 male; mean age: 24.52 years; SD: 4.92 years) the participants were randomly allocated to one of two groups of *n* = 23 participants each, which encountered different emotional pictures (faces or scenes, see below) in a between-group design. the sample size was supported by a priori power analysis, based on a mean cohen's *d* = 0.48 for a context × emotion valence interaction effect that was based on the effects of previous studies that investigated emotional modulations of contextual cueing (Kunar et al., 2014; Meyer et al., 2019; Zinchenko et al., 2020). accordingly, a sample size of *n* = 46 participants was appropriate to detect an effect size with 89% power using the R library “webpower” (Zhang et al., 2018).

All participants reported normal or corrected-to-normal vision and were naïve as to the purpose of the study. The experimental procedure was in accordance with the Declaration of Helsinki and approved by the Ethics Committee of the Department of Psychology at Ludwig-Maximilians-Universität München. Participants provided written informed consent before the experiment and received either course credit or payment of 9 euros (~10 USD). All participants reported normal or corrected-to-normal vision.

### Stimuli and procedure

The experiment was programmed using Matlab with Psychtoolbox extensions (Brainard, 1997; Pelli, 1997). Participants sat in a dimly lit laboratory in front of a 23-inch LCD monitor (ASUS, Taiwan; 60 Hz refresh rate; 1,920 × 1,080 pixels resolution) at a viewing distance of approximately 80 cm. Search displays contained 12 black items (1.00 cd/m^2^; size: 0.35° × 0.35°) presented on a white screen background (25.40 cd/m^2^). There were 11 L-shaped distractor items which were presented at orthogonal orientations (0°, 90°, 180°, or 270°), and one T-shaped target item, which was rotated by either 90° or 270°. Items were placed on four imaginary concentric circles around the center of the display, with radii of 1.74°, 3.48°, 5.22°, and 6.96°, respectively. Items were positioned so that at least three items were in each display quadrant. Target locations (in repeated and non-repeated displays) were pseudo-randomized such that targets appeared equally often on ring 2 vs. ring 3, the left vs. right display half, and the upper vs. lower display half. There were 24 potential target locations, from which 12 were used for repeated displays (with fixed distractor layout) and 12 for non-repeated displays (with random distractor arrangements).

Emotional images were presented for 500 ms at variable (50, 500, 1,000 ms) intervals before the search displays (cf. Bocanegra and Zeelenberg, 2009). The two groups of participants either encountered faces from the NimStim Database (Tottenham et al., 2009) or they were presented with IAPS scenes (Lang et al., 1997). The participants in the face group saw images with angry, fearful, disgusting, and neutral facial expressions of diverse genders and races (see Appendix). A total of 36 negative and 36 neutral facial expressions were shown. The identity of facial expressions was the same—once negative and once neutral. The images were randomly selected, but it was assured that the following conditions were fulfilled: (1) an equal number of male and female faces, (2) selected face identities containing images of both negative and neutral emotional expressions, (3) there was a diversity in the racial composition of images. Participants in the IAPS group also saw 36 negative pictures conveying strong feelings of either sadness or disgust and 36 neutral pictures (see Appendix). We chose sadness and disgust because the International Affective Picture System provides a particularly large number of pictures (>12) that elicit these discrete emotions (Mikels et al., 2005).

### Search task

Each trial started with a presentation of an emotional or neutral picture for 500 ms, followed by a blank stimulus interval of 50, 500, and 1,000 ms, randomized across trials. Next, the search arrays containing a T target letter and 11 distractor letters were presented. Each display was presented until observers made a speeded response (by pressing the right or left arrow key of the computer keyboard depending on the corresponding left/right orientation of the target). A blank screen of 500 ms followed in case of a correct responses. In case of a response error, a warning message (“Wrong”) appeared on the screen for 1,000 ms, which was followed by another (blank) interval of 1,000 ms until the next trial began. The visual search task comprised 864 trials, divided into 36 blocks of 24 trials each. In each block, there were 12 repeated displays together with 12 non-repeated displays. Repeated displays had fixed distractor locations, while the layout of distractors in non-repeated displays was generated anew on each trial. Targets appeared at a fixed set of 24 locations throughout the experiment: 12 locations were used for repeated displays, and 12 (other) locations for non-repeated displays. With the latter, we controlled for target location repetition effects across the two types of displays. Thus, any beneficial effects arising from repeated displays could only be attributed to the effects of repeated distractor-target contexts rather than absolute target positions in these displays.

**Figure 1:**
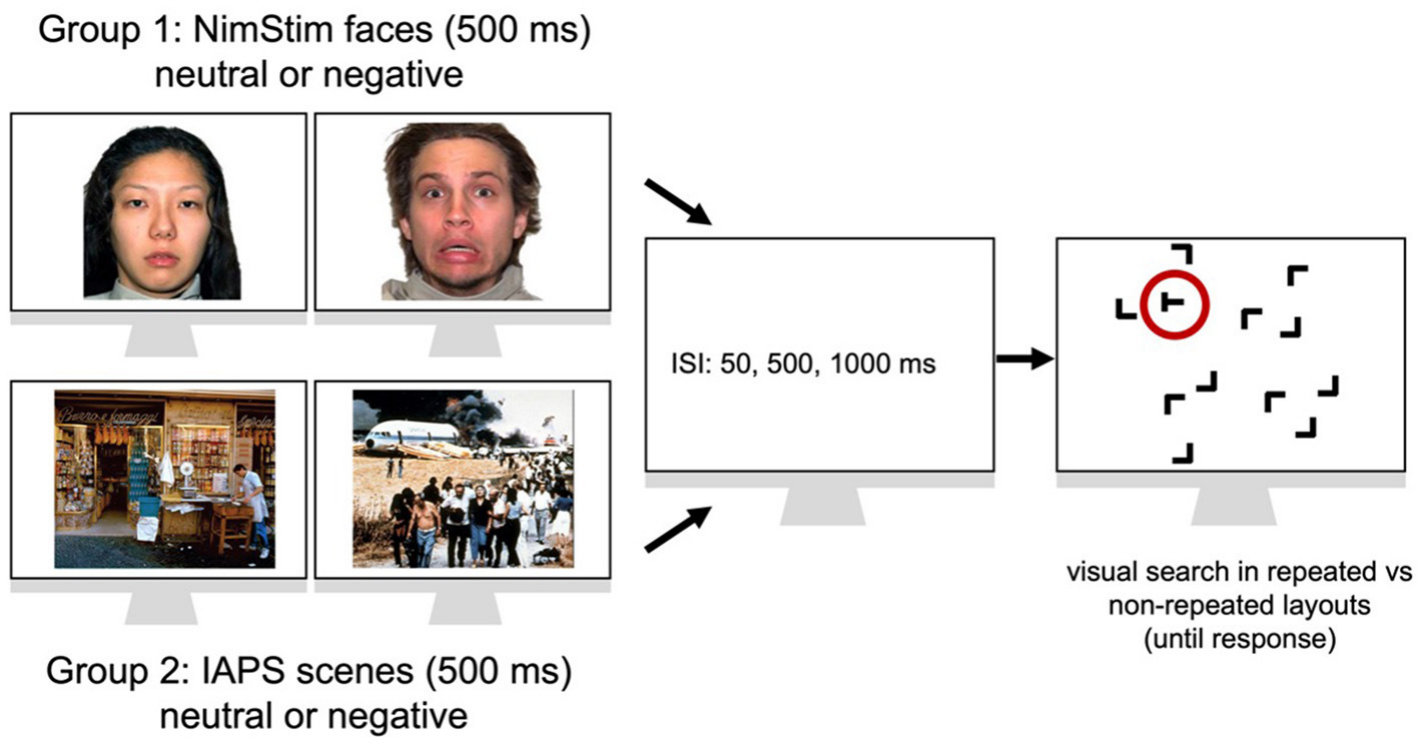
Two independent groups of *N* = 23 participants each encountered neutral vs. emotional faces from either the NimStim database (group 1) or experienced neutral vs. emotional scenes from the International Affective Picture System (group 2). For each group, an emotional or a neutral stimulus was shown for 500 ms and followed by a blank screen of 50, 500, or 1,000 ms (inter stimulus interval: ISI) until the search displays containing one target T letter (marked by a red circle, which is only shown for illustrative purposes) and 11 distractor L letters. The participant's task was to discriminate the left- vs. right-oriented T target letter (in the example, the target is left-oriented). Unbeknown to them, half of the trials contained repeated target-distractor layouts with fixed target and distractor locations, while the other half had non-repeated, i.e., random, arrangements of target and distractor items. Face stimuli sourced from the NimStim set of Facial Expressions (Tottenham et al., 2009) and available to the scientific community at https://macbrain.org/resources/. All faces are from the Nim Stim data set publicly available for scientific research.

Further, each block had 12 emotional and 12 neutral pictures (randomly chosen from our total set of NimStim/IAPS pictures presented in the Appendix), with the restriction that emotional pictures were evenly distributed, containing four angry, four fearful, and four disgusting expressions in each block of the face group, while there were six pictures associated with sadness and six pictures associated with disgust in each block of the IAPS group. Each block (of 24 trials) had six trials with repeated displays presented together with emotional stimuli, six trials with repeated displays presented together with neutral pictures, six trials with non-repeated layouts and emotional pictures and six trials with non-repeated displays presented together with neutral pictures. Thus, emotional and neutral pictures were shown together with both repeated and non-repeated displays. Any processing differences between the two types of displays are thus unlikely to be attributable to emotional or neutral picture composition differences. Distinct sets of non-/repeated displays were presented together with either emotional or neutral pictures (that were nevertheless randomly chosen from our NimStim/IAPS picture set, thus preventing participants from learning specific pairings between individual emotional/neutral pictures and individual search layouts). We were thus able to test how emotions influence the cueing effect by analyzing RTs to (a set of) repeated and non-repeated configurations that were always paired with emotional images and RTs to (a different set of) repeated and non-repeated layouts that were always presented together with neutral images. Figure 1 shows the corresponding stimulus design and sequence of trials.

## Results

For the main analysis, errors and outliers (RTs ± 2.5 SD from the mean, comprising <1% of all trials) were excluded before conducting a 2 × 2 × 2 × 3 × 3 mixed-design analysis of variance (ANOVA) on the averaged RTs across all 36 blocks. The independent factors in this analysis were: (1) emotional stimulus (face, scene; between-subject factor); (2) emotional valence (negative, neutral; within-subject factor); (3) search context (repeated, non-repeated; within-subject factor); (4) epoch (1–3; within-subject factor; one epoch contained data from 12 blocks to obtain stable estimates of our measurements); and, (5) inter-stimulus interval (ISI) between the presentation of the face/scene stimulus and search display (50, 500, or 1,000 ms; within-subject factor). We also conducted exploratory analyses to better understand the patterns within the (main) data as well as to explore potential alternative explanations of the data. Specifically, we tested qualitatively whether our effect pattern was due to individual differences by computing the contextual-cueing effect for each participant and each emotional valence (negative, neutral) and emotional stimulus (face, scene). Also, within-participant comparisons were made by counting the number of participants exhibiting greater contextual cueing for neutral vs. emotional stimuli for faces and scenes. Additional exploratory analyses were conducted by quantitatively analyzing RTs for only subsets of trials containing identical emotional events, such as disgusting faces vs. disgusting scenes, using paired-sample *t*-tests.

### Main analysis

Repeated displays elicited faster RTs than non-repeated displays, an effect that became more pronounced as the experiment progressed, *F*_(1,44)_ = 13.58, *p* < 0.001, $\eta_{\text{p}}^{2}$ = 0.35 (significant context x epoch interaction). The magnitude of contextual cueing (RTs in non-repeated minus RTs in repeated displays) in the first epoch of the experiment was 17 ms, which compares to 63 ms in the second epoch, *t*_(45)_ = −4.74, *p* < 0.001, 95% CI (−65.26 ms, −26.51 ms), and 60 ms in the third epoch, *t*_(45)_ = −3.75, *p* < 0.001, 95% CI (−66.26 ms, −19.97 ms).

Moreover, contextual cueing differed between emotional and neutral pictures, though these effects were dependent on the type of affective stimulus used, *F*_(1,44)_ = 6.46, *p* < 0.001, $\eta_{\text{p}}^{2}$ = 0.13 (significant context x emotional stimulus x emotional valence interaction): for participants in the face group, the mean contextual-cueing effect was 70 ms for faces with neutral expressions, which compares to a cueing effect of 46 ms for negative faces *t*_(22)_ = −1.27, *p* = 0.21, 95% CI (−15.24 ms, 63.69 ms). This was different from participants in the scene group, where cueing was absent with neutral images (−1 ms), thus contrasting with a strong cueing effect of 72 ms with negative images, *t*_(22)_ = −2.20, *p* < 0.05, 95% CI (−142.42 ms, −4.22 ms). The cueing effects of 70, 46, and 72 ms were all reliable (*t*'s > 2.90, *p*'s < 0.0083).

**Figure 2:**
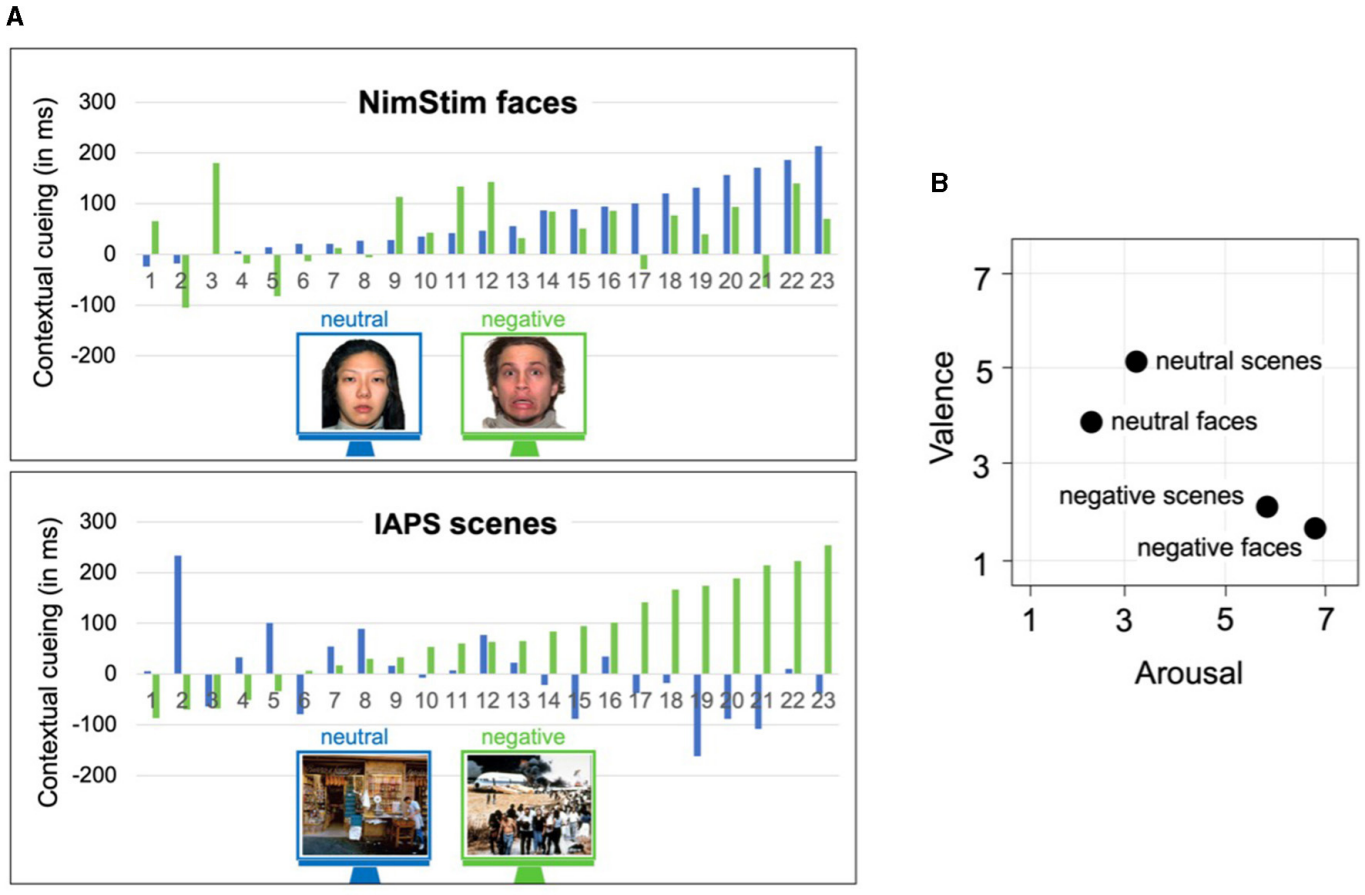
Result from the main analysis. **(A)** Displays search RTs in ms in repeated and non-repeated layouts (green and gray lines, respectively) for neutral vs. emotional NimStim faces in the upper panel and neutral vs. emotional IAPS scenes in the lower panel. Separate graphs are additionally depicted for the various intervals (ISI: 50, 500, 1,000 ms) separating the face/scene images from the search displays. **(B)** Reveals the mean contextual-cueing effects: RTs in non-repeated displays minus RTs in repeated displays for neutral vs. emotional NimStim faces **(top)** and IAPS scenes **(bottom)**. Face stimuli sourced from the NimStim set of Facial Expressions (Tottenham et al., 2009) and available to the scientific community at https://macbrain.org/resources/. All faces are from the Nim Stim data set publicly available for scientific research.

Contextual cueing also increased with increasing temporal distance between the emotional stimulus and the search displays, *F*_(1,44)_ = 6.25, *p* < 0.01, $\eta_{\text{p}}^{2}$ = 0.12 (; significant context × ISI interaction). Contextual-cueing effects with a preceding 50, 500, and 1,000 ms ISI were 33, 48, 59 ms, respectively, though this effect was not systematically influenced by emotional valence or emotional stimulus type (*F*'s < 1.25, *p*'s > 0.26). Figure 2 depicts how RTs to repeated and non-repeated displays developed with emotional and neutral images for the two groups.

### Exploratory analysis

**Figure 3:**
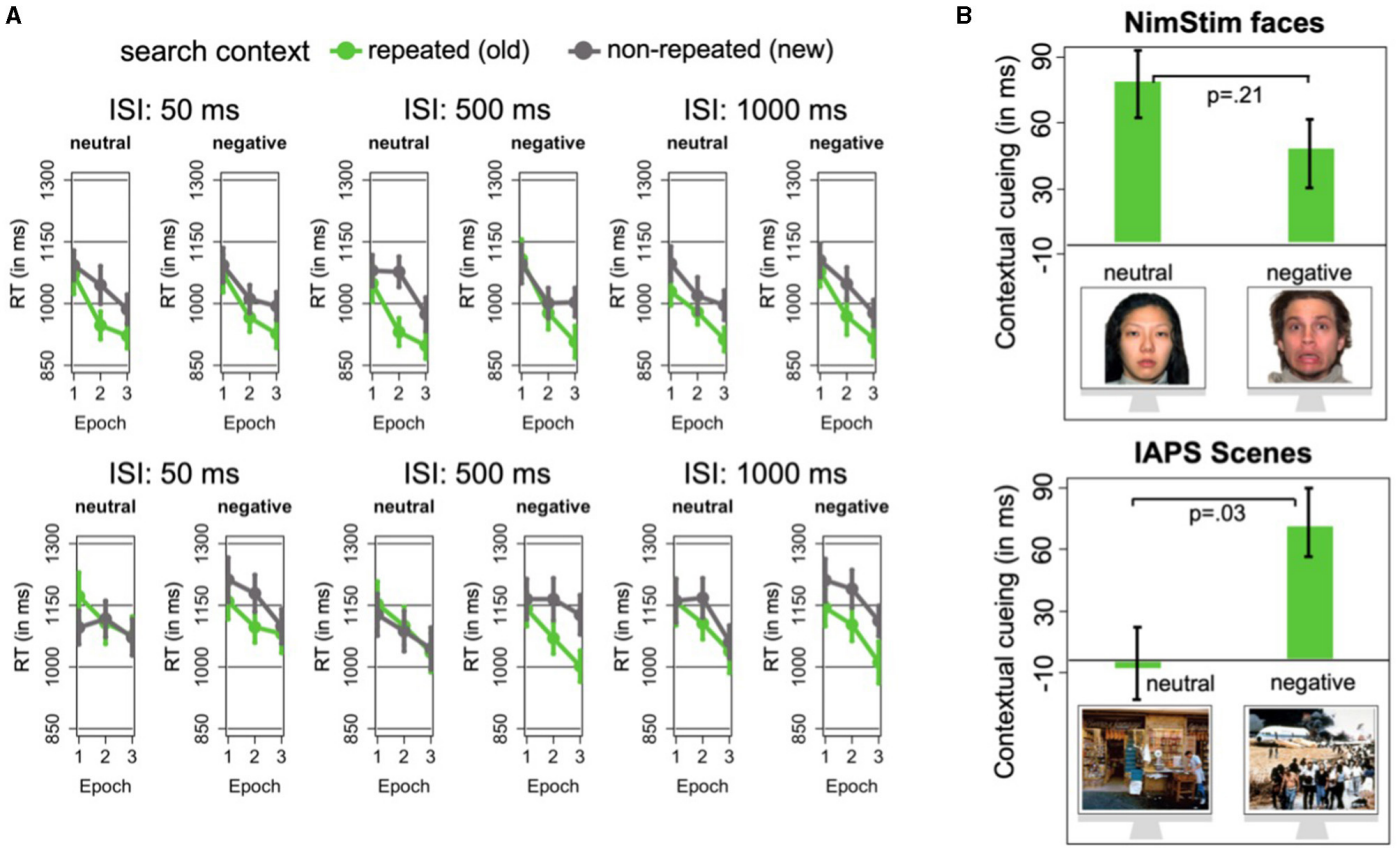
Results from exploratory analysis. **(A)** Shows the contextual cueing effects (RTs in non-repeated displays minus RTs in repeated displays) for each individual participant in the face **(top)** and scene **(bottom)** groups for the neutral and negative emotional expressions (blue and green bars, respectively). For the face group, participants are rank ordered by the size of their contextual-cueing effects with neutral faces. For the scene group, the participants are sorted according to the individual contextual-cueing effects with negative scenes. **(B)** The mean arousal and valence scores for the face and scene stimuli in a common arousal *x* valence space (*x*- and *y*-axes, respectively), with these (independent) ratings coming from prior studies (Lang et al., 1997; Sutton et al., 2019) that used identical self-assessment manikin scales to evaluate the arousal and valence levels of the scene/face stimuli. Face stimuli sourced from the NimStim set of Facial Expressions (Tottenham et al., 2009) and available to the scientific community at https://macbrain.org/resources/. All faces are from the Nim Stim data set publicly available for scientific research.

Thus far, our analyses revealed that negative facial expressions did not affect the cueing effect—if anything, the RT facilitation for the repeated layouts was weaker with emotional than neutral faces. This contrasts with contextual cueing in scenes, where the cueing effect was increased and significant only with emotional (relative to neutral) scenes. As shown in Figure 3, these differences were also reflected at the individual participant level. For instance, 16 out of 23 participants showed a reliable (i.e., greater than zero) contextual-cueing effect with emotional faces, which compares to 20 out of 23 participants who displayed a cueing effect with neutral faces. When the search displays were presented with emotional scenes, a cueing effect was found for 18 out of 23 participants, contrasting with only 12 out of 23 participants who developed a cueing effect with neutral scenes. Notably, this pattern remains even when comparing the cueing effect between emotional and neutral pictures within each participant. For the face group, there were 17 out of 23 participants with greater contextual cueing with neutral compared to emotional faces. This was again reversed for the scene group, in which 15 out of 23 participants displayed increased cueing with emotion over neutral scenes. These results thus suggest that emotions modulate contextual learning at the individual-participant level. Moreover, these findings additionally suggest that the effect of emotions upon contextual cueing crucially depends on the stimulus material: contextual learning is somewhat weaker in individual participants that experience emotional (vs. neutral) faces, while the learning of repeated layouts is conversely more pronounced, and reliable only, in individual observers that encounter emotional (vs. neutral) scenes.

Next, we tested whether emotional arousal may explain this opposing result pattern for scenes vs. faces. For instance, it was suggested that emotional experiences can be described along the dimensions of valence and arousal (though these dimensions often co-vary; see, e.g., Zsidó, 2024). An emotional modulation of contextual learning might thus arise because of variations in the arousal level of our stimuli (independent of their valence). However, an analysis of our stimuli's arousal (and valence) levels did not support this hypothesis (see Figure 3B). We found that neutral faces and scenes were associated with lower arousal scores (2.51 and 3.32, respectively) compared to faces and scenes conveying negative emotions (6.81 and 5.82, respectively). A similar pattern was obtained for valence: neutral faces and neutral scenes had overall higher valence scores (3.72 and 5.09, respectively) compared to negative faces and negative scenes (1.51 and 2.31). However, emotional modulation of contextual learning only emerged for scenes, but not for faces: contextual cueing was larger for negative (72 ms) than neutral (-1 ms) scenes, but the direction of the cueing effect was reversed for faces, revealing a larger effect for neutral (70 ms) as compared to negative (46 ms) faces.

Thus, these results show that variations in emotional arousal (and valence) are unlikely to explain the pattern of contextual learning effects across face and scene stimuli. Noteworthy, both faces and scenes were normed using an identical—self-assessment manikin—scale on the dimensions of arousal and valence (IAPS scenes: Lang et al., 1997; NimStim faces: Sutton et al., 2019), thus permitting us to directly compare our face and scene stimuli in terms of their arousal and valence scores.

We went on to examine whether differences in contextual learning between faces and scenes are attributable to differences in the type of emotional event that was signaled by our stimuli. For instance, discrete emotions of fear, anger, and disgust elicited by our NimStim faces may lead to qualitatively different processing relative to the experiences of disgust and sadness induced by our IAPS scenes (see Mikels et al., 2005). Given this, we re-analyzed the RTs by considering only subsets of trials that had identical emotional events: disgusting NimStim faces and disgusting IAPS scenes. Despite identical discrete emotions, we still observed opposing effects in our data: contextual cueing was (numerically) *stronger* with neutral relative to emotional faces that display disgust [76 and 50 ms, respectively; *t*_(22)_ = 1.31, *p* = 0.201], while the opposite was true for scenes, where the cueing effect was reduced for neutral relative to emotional disgust-inducing images [−1 and 72 ms, respectively; *t*_(22)_ = −1.99, *p* = 0.029], thus mirroring the overall pattern of results as described above.

In sum, we found an interaction such that contextual learning was increased, and reliable only, with emotional compared to neutral scenes, while the contextual RT facilitation was statistically comparable between emotional and neutral faces. Additional analyses showed that emotional images modulate contextual learning at the individual participant level and that differences in contextual learning between face and scene pictures are unlikely to be due to differences in the concrete emotions elicit by our stimuli or their arousal/valence levels. Accordingly, we suspect that the dissociation in contextual learning between faces and scenes is due to other factors including, e.g., differences in how individual participants perform the search task in terms of a more active vs. more passive deployment of attention in the search displays, which, in turn, should influence contextual learning (Lleras and Von Mühlenen, 2004).

### Recognition performance

Participants' explicit recognition performance was assessed by their ability to correctly recognize repeated displays (“hits”) as compared to their incorrect classification of non-repeated displays as repeated (“false alarms”). Hits and false alarms were entered into a 2 (emotional stimulus: faces vs. scenes) × 2 (emotional valence: negative vs. neutral) × 2 (response type: hits vs. false alarms) mixed-design ANOVA, which revealed a significant main effect for response type, *F*_(1,44)_ = 11.13, *p* = 0.002, $\eta_{\text{p}}^{2}$ = 0.20, indicating that participants were more likely to correctly identify a repeated context as repeated (hit rate: 58.2%), relative to incorrectly identifying a non-repeated display as repeated (false alarm rate: 48.6%). No other main effects or interactions reached statistical significance (*F*'s < 1.91, *p*'s > 0.17). These results, thus, suggest that contextual cueing is, at least to some extent, accessible to awareness (e.g., Smyth and Shanks, 2008; Vadillo et al., 2016; Geyer et al., 2020; Meyen et al., 2023).

## Discussion

Our study had two main goals. First, we aimed to investigate the emotional modulation of statistical learning of target-distractor co-occurrences in search tasks when emotional events were presented at variable intervals before the search task. Second, by using behaviorally relevant face and scene stimuli, we wanted to see whether these stimuli differed in their capacity to modulate statistical learning in visual search. We found that search in repeated contexts was overall more effective than search in non-repeated displays (containing random target-distractor layouts), thus revealing a typical contextual-cueing effect. Crucially, we found that contextual facilitation of search was increased for emotional compared to neutral scenes, while no such an effect was observed for faces, where negative faces elicited an even numerically smaller contextual-facilitation effect (compared to neutral faces). This interaction was seen at all intervals separating the emotional/neutral images from the search displays. Moreover, differences in mean contextual cueing were also observed at the individual participant level and these differences were unlikely to be due to variability in the arousal/valence levels or related to discrete emotions signaled by our face and scene pictures. But we acknowledge that the arousal and valence ratings for the face and scene stimuli may not be fully comparable. This is because these ratings were collected from different samples of participants (and obtained at different times: IAPS: 2008; NimStim: 2019). Therefore, future work may be carried out in which individual participants evaluate both face and scene stimuli to better compare the arousal and valence ratings across image categories.

We take our results to mean that emotion-inducing images presented before the search displays influence how participants perform the (rather) difficult search task. In this task, all search items are composed from the same line elements, thus providing rather limited opportunities for bottom-up guidance, which would improve search (Moran et al., 2013). Accordingly, participants may need to solve the task by scrutinizing individual search items until they arrive at the target location. This effortful scanning strategy contrasts with passive search, during which participants would allow that the unique target item (eventually) pops into their attention focus (Lleras and Von Mühlenen, 2004). Importantly, such a passive search strategy may also facilitate contextual learning, where the target is prioritized by acquired target-distractor contextual memories. If we assume that visual search can be either active or passive, then emotional experiences may alter the relative interplay between these opposing strategies. For instance, emotional images may induce a bias to perform visual search in more passive mode. As such, emotional events would complement the effects found in previous studies in which participants received explicit instructions to perform the visual search actively or passively (e.g., Lleras and Von Mühlenen, 2004; Watson et al., 2010; see also Smilek et al., 2006). Our account is also in line with several previous findings:

For instance, it was reported that negative emotional events engage selective attention in an automatic, i.e., involuntary, manner and thus receive priority in sensory processing (e.g., Öhman et al., 2001; Vuilleumier, 2005; Bradley, 2009). Moreover, while attentional resources are limited (e.g., Desimone and Duncan, 1995; Müller et al., 1995), an active search strategy may likewise consume attentional processing resources. During such an attention-demanding search task, negative emotions could bias processing from an active—toward a more passive search (because the emotions withdraw attentional resources and thus reduce active search), which would also facilitate incidental, i.e., automatic, learning of statistical regularities.

Further evidence for a modulation of sensory processing by emotional scene and face stimuli has been reported in EEG studies, which showed that specific components in the N1/P2 latency range are more pronounced for emotional relative to neutral stimuli (N170 and EPN components; see, e.g., Thom et al., 2014; see also Zinchenko et al., 2015, 2017). Prioritization of emotional events may nevertheless differ between face and scene stimuli. For example, Eimer and Holmes (2007) reported evidence that the N170 emotional face effect vanishes when participants perform a concurrent attention-demanding task. Moreover, Thom et al. (2014) reported increased EPN waveforms for emotional scenes compared to emotional faces. This latter finding may thus suggest that emotional scenes are more effective in automatically capturing attention. In this view, emotional scenes may carry greater weight in biasing individual participants toward passive search, thus in turn facilitating contextual learning. This view is supported by findings showing that during the recognition of emotional faces, participants often inspect the eye and mouth regions (as measured with eyetracking; e.g., Eisenbarth and Alpers, 2011). Decoding of emotional face information may thus engage top-down attention (toward the eye/mouth region), which may also bias active search toward individual display items/regions. As a result, statistical learning could be reduced with negative compared to neutral faces as we have observed in the present investigation (albeit not significantly so).

In the current study, contextual cueing was increased with emotional compared to neutral scenes, a result which aligns well with previous investigations of the cueing effect; although these studies used different emotion induction schemes, where emotional images were presented together with the search displays (e.g., Meyer et al., 2019; Zinchenko et al., 2020). Given that emotions can induce transient vs. sustained effects (e.g., Qiao-Tasserit et al., 2017), then our result might reflect a rather slow, yet transient emotion effect, where negative scenes influence visual search within shorter and somewhat longer durations (50–1,000 ms in the present investigation) and up-modulate statistical context learning in this period. This could also explain why our results deviate from other investigations, in which the cueing effect was even weaker with emotional pictures (e.g., Kunar et al., 2014), and where participants viewed the emotional pictures in a block before the actual search task. This latter manipulation could thus have recruited a different form of—sustained—emotional engagement, or a change in mood, which may have led to different behavioral and cognitive processing than the induction of emotions at rather short temporal windows in the current study.

Taken together, our results offer new insights into how emotions may influence visual search. Emotional stimuli involuntarily withdraw processing resources and modulate behavioral and electrophysiological indices in visual search (e.g., Hindi Attar and Müller, 2012). This contrasts with the beneficial effects of repeated search layouts: Searching a repeated display triggers learning about where to find the search target, leading to the acquisition of contextual search templates in long-term memory (e.g., Brady and Chun, 2007) that point to the target position within the spatial context of distractor items. Activation of such a contextual template by a given repeated search display renders target localization (and, thus, responses to the target) more efficient compared to non-repeated (never encountered) displays—constituting the contextual-cueing effect (Chun and Jiang, 1998). The current study shows that an emotional up-modulation of the cueing effect occurs with scenes but not faces, thus potentially “sharpening” the contextual search template and thereby improving context-based guidance by induced emotional contents (at least when appropriate, emotion-inducing stimuli are presented). We acknowledge that our study “only” considered emotional faces and scenes, so future research using, e.g., auditory stimuli like sounds or music clips to evoke certain emotions could be a complementary method to study the emotional variation of statistical learning in search tasks. Participants may also read brief narratives or view short video clips to elicit specific emotions, which could eventually better approximate emotional experiences in real-world situations. Broadening the scope of stimuli would increase the generalizability of the present findings. It could reveal similarities and differences in how distinct types of emotional stimuli modulate processes like contextual cueing.

## Conclusion

In conclusion, our findings provide new evidence that emotional scenes improve statistical learning of target-distractor contingencies in visual search. We propose that emotional scenes do so by withdrawing attentional resources, biasing participants to perform visual search in a passive, i.e., receptive, mode, which improves contextual learning and thereby enhances the contextual-cueing effect. These results thus advance our understanding of how specific emotional events influence visual statistical learning: while emotional images capture attention and processing resources, this does not necessarily impede statistical learning, which, by default, operates automatically. Emotional biases could then be considered as a mechanism that adjusts the relative interplay of automatic and controlled processing in search tasks.

## Data availability statement

The raw data supporting the conclusions of this article will be made available by the authors, without undue reservation.

## Ethics statement

The studies involving humans were approved by Ethics Committee of the LMU, Munich. The studies were conducted in accordance with the local legislation and institutional requirements. The participants provided their written informed consent to participate in this study.

## Author contributions

AZ: Writing—review & editing, Writing—original draft, Project administration, Methodology, Investigation, Formal analysis, Data curation, Conceptualization. AB: Writing—review & editing, Investigation, Formal analysis, Data curation, Conceptualization. MC: Writing—review & editing, Supervision. HM: Writing—review & editing, Supervision, Resources, Conceptualization. TG: Writing—review & editing, Writing—original draft, Visualization, Validation, Supervision, Resources, Funding acquisition, Formal analysis, Data curation.

## Appendix

**Table A1:** List of NimStim images presented in the experiment.

**Emotion**	**Image Code**
Neutral	01F_NE_C, 02F_NE_C, 03F_NE_C
	05F_NE_C, 06F_NE_C, 07F_NE_C
	08F_NE_C, 09F_NE_C, 10F_NE_C
	11F_NE_C, 12F_NE_C, 13F_NE_C
	14F_NE_C, 15F_NE_C, 16F_NE_C
	17F_NE_C, 18F_NE_C, 19F_NE_C
	20M_NE_C, 21M_NE_C, 22M_NE_C
	23M_NE_C, 24M_NE_C, 25M_NE_C
	26M_NE_C, 27M_NE_C, 28M_NE_C
	29M_NE_C, 30M_NE_C, 31M_NE_C
	32M_NE_C, 33M_NE_C, 38M_NE_C
	39M_NE_C, 40M_NE_C, 41M_NE_C
Anger	08F_AN_O, 09F_AN_O, 10F_AN_O
	14F_AN_O, 18F_AN_O, 19F_AN_O
	29M_AN_O, 31M_AN_O, 32M_AN_O
	34M_AN_O, 42M_AN_O, 43M_AN_O
Disgust	05F_DI_O, 06F_DI_O, 07F_DI_O
	13F_DI_O, 16F_DI_O, 17F_DI_O
	24M_DI_O, 25M_DI_O, 26M_DI_O
	27M_DI_O, 28M_DI_O, 40M_DI_O
Fear	01F_FE_O, 02F_FE_O, 03F_FE_O
	11F_FE_O, 12F_FE_O, 15F_FE_O
	20M_FE_O, 21M_FE_O, 22M_FE_O
	23M_FE_O, 38M_FE_O, 39M_FE_O
Neutral	1350, 1390, 2038, 2102, 2190
	2191, 2210, 2235, 2305, 2575
	5395, 5471, 5532, 5740, 6150
	7000, 7002, 7004, 7006, 7010
	7055, 7057, 7059, 7061, 7090
	7207, 7233, 7235, 7242, 7491
	7500, 7547, 7550, 7590, 7595
	7640
Sad	2053, 2205, 2276, 2800, 2900
	3230, 3301, 3350, 9041, 9050
	9252, 9410, 9415, 9421, 9520
	9561, 9611, 9921
Disgust	2352.2, 3010, 3071, 3080, 3110
	3120, 3130, 3250, 3400, 7361
	8230, 9042, 9290, 9300, 9320
	9405, 9490, 9570
